# A multicentric randomized controlled trial on the impact of lengthening the interval between neoadjuvant radiochemotherapy and surgery on complete pathological response in rectal cancer (GRECCAR-6 trial): rationale and design

**DOI:** 10.1186/1471-2407-13-417

**Published:** 2013-09-12

**Authors:** Jérémie H Lefevre, Alexandra Rousseau, Magali Svrcek, Yann Parc, Tabassome Simon, Emmanuel Tiret

**Affiliations:** 1 Assistance Publique-Hôpitaux de Paris (AP-HP), Department of Digestive Surgery, Hôpital Saint-Antoine, 184, rue du Fbg Saint-Antoine, 75012 Paris, France; 2 University Pierre & Marie Curie (UPMC-Paris 06), Paris, France; 3 AP-HP, Clinical Research Unit (URC-Est), Department of Clinical Pharmacology, Hôpital Saint-Antoine, Paris, France; 4 AP-HP, Departement of pathology, Hôpital Saint-Antoine, Paris, France

**Keywords:** Rectal cancer, Radiochemotherapy, Complete histological response, Procedure

## Abstract

**Background:**

Neoadjuvant radiochemotherapy (RCT) is now part of the armamentarium of cancer of the lower and middle rectum. It is recommended in current clinical practice prior to surgical excision if the lesion is classified T3/T4 or N+. Histological complete response, defined by the absence of persistent tumor cell invasion and lymph node (ypT0N0) after pathological examination of surgical specimen has been shown to be an independent prognostic factor of overall survival and disease-free survival. Surgical excision is usually performed between 6 and 8 weeks after completion of CRT and pathological complete response rate ranges around 12%. In retrospective studies, a lengthening of the interval after RCT beyond 10 weeks was found as an independent factor increasing the rate of pathological complete response (between 26% and 31%), with a longer disease-free survival and without increasing the operative morbidity. The aim of the present study is to evaluate in 264 patients the rate of pathological complete response rate of rectal cancer after RCT by lengthening the time between RCT and surgery.

**Methods/design:**

The current study is a multicenter randomized trial in two parallel groups comparing 7 and 11 weeks of delay between the end of RCT and cancer surgery of rectal tumors.

At the end of the RCT, surgery is planified and randomization is performed after patient’s written consent for participation. The histological complete response (ypT0N0) will be determined with analysis of the complete residual tumor and double reading by two pathologists blinded of the group of inclusion. Patients will be followed in clinics for 5 years after surgery. Participation in this trial does not change patient’s management in terms of treatment, investigations or visits. Secondary endpoints will include overall and disease free survival, rate of sphincter conservation and quality of mesorectal excision. The number of patients needed is 264.

**Trial registration:**

ClinicalTrial.gov: NCT01648894

## Background

Rectal cancer represents 12 000 new cases each year in France [1]. Its management is based on a multidisciplinary management with radiotherapy, chemotherapy and surgery.

### Surgery

Surgery consisting of total excision of the mesorectum has improved over time. The studies of Heald [2,3] have showed that local recurrence rate was directly related to the preservation of the fascia recti during the dissection of the rectum. In countries where this surgical procedure was emphasized, the local recurrence rate and life expectancy after treatment of rectal cancer were improved [4,5]. In Sweden, after learning of the mesorectal excision technique, 447 patients were compared to older cohorts, the local recurrence rate at 5 years was increased from 20.5% to 8.2% and 5-year survival of 65.8% to 77.3% (p < 0.001 for both comparisons).

### Radiotherapy

Before the widespread diffusion of the technique of mesorectal excision, many studies, including 4 randomized studies, had compared pre-operative radiotherapy (RT) versus surgery alone [6-9]. Results showed only a benefit in terms of local control with a reduction of 10% to 5% of the recurrence rate local for patients with optimal dissection of the mesorectum, but no survival benefit. In 1997, a randomized study in 1168 subjects showed an improved survival with neoadjuvant RT: survival at 9 years was 65% for patients with no radiotherapy compared to 74% for patients with neoadjuvant RT (p = 0.002) [9]. Local recurrence for patients treated by surgery alone was also more frequent (27% (150/557) compared to 11% (63/553), p < 0.001) [9]. This finding was explained by a suboptimal surgical resection without complete mesorectum excision. As a consequence, an international randomized trial study comparing RT + surgery versus surgery alone with quality control of excision mesorectum [10] was conducted in 1805 patients. The results confirmed a higher rate of local control at 2 years in patients with preoperative radiotherapy (2.4% vs. 8.3%, p < 0.001), with no difference in terms of survival (82% vs. 81.8%, p = 0.84). Early postoperative complications were higher in the RT + surgery group (48% *vs*. 41%, p < 0.01) [11]. After a minimum of 5 years of follow-up, long-term complications; were more frequent in the preoperative radiotherapy group with (62% and 56% versus 38% and 33% of episodes of incontinence and pad wearing respectively; p < 0.001) [12]. As postoperative morbidity and results functional obtained after preoperative RT were very significantly altered, the authors recommended such treatment only in patients at high risk of local recurrence. These recommendations were proposed by the French experts for the treatment of rectal cancer and were approved by the *Haute Autorité de Santé* in 2007 [13].

### Chemoradiotherapy

Other studies have evaluated the impact of adding chemotherapy to RT [14,15]. The randomized trial of Bosset et al. included between 1993 and 2003, 1011 patients with rectal cancer T3 or T4 and analysed the impact of addition of chemotherapy based on 5-fluorouracil to RT 45Gy [14] compared with preoperative RT alone. The complete response rate (pT0) on resected specimen was 5.3% in the RT alone group and 13.7% in the chemoradiotherapy (CRT) group (odds ratio = 2.84, 95%CI: 1.75 to 4.59, p < 0.0001) [15]. No benefit in overall survival or disease-free survival was observed in CRT group despite a significant improvement rate of local recurrence at 5 years (8.7% in the CRT compared to 17.1% after RT alone *vs*., p = 0.002). However, the results cannot be generalized because of the lack of uniform total mesorectal excision for all patients included in this study.

However, CRT has allowed the team of Pr. Habr-Gama to obtain a complete clinical regression of tumor rectal in 26.8% of cases (71/265 patients) [16]. The protocol consisted of 50 Gy radiation therapy combined with chemotherapy based on 5-fluorouracil and leucovorin. This group of patients had not been operated and simple surveillance without resection was performed. After a mean follow-up of 57 months, only 2 patients (2.8%) had local recurrence and 3 had distant metastases. The 5-year recurrence rate was 7%. The complete clinical response was associated with better overall survival at 5 years: 100% versus 88% (p = 0.01).

In a retrospective series of the Cleveland Clinic, among 238 patients treated with neoadjuvant CRT, 58 (24.4%) had a ypT0N0 tumor on pathological exam. Postoperative morbidity was similar in the neoadjuvant CRT group and in the no-pCR group, but there was a better local control (5 years local recurrence rate: 0% *vs*. 10.6%, p < 0.001) [17].

### Complete response: the importance of the period between the end of radiotherapy and surgery

The median complete response rate is about 12% [7-27%] in the main series of rectal cancer treated with RT or RCT [18-31]. A randomized study in 1999 had already found an effect in tumor reduction after RT between two intervals (2 weeks or 7 weeks) clinical response increased from 53.1% to 71.7% (p = 0.007) and pCR or near complete (persistence of some tumor cells) increased from 10.3% to 26% (p = 0.0054) [21]. The results of this study cannot be generalized, considering the use of a technique of mesorectal excision other than the widely distributed and administered neoadjuvant therapy (RT alone of 40 Gy). However the interest of extending the period after RT was obvious. In 2003, Moore’s team showed that among 155 rectal cancers treated with neo-adjuvant RCT (50 Gy + 5-fluorouracil), the rate of complete response increased from 9% to 23% between patients operated on before the 40^th^ day (between 6^th^ and 7^th^ week) and those operated on after waiting more than 7 weeks (p = 0.09) [27].

Following the work of Prof. Habr-Gama, other authors have attempted to identify factors associated with a pCR after surgical resection. The first study published by Tulchinsky et al. [29] in 2008 reported the retrospective findings on the time between preoperative CRT (45–50 Gy + 5-fluorouracil) and surgery (less or more than 7 weeks) in 132 patients operated on by anterior resection or abdomino-perineal resection between 2000 et 2006. The rate of pCR or near complete (persistence of microscopic foci of adenocarcinoma in the rectal wall without lymph node) was 28% in the resected specimen. The single independent factor associated with a good response was the period between the end of RT and surgery: 17% in the group operated <7 weeks against 35% in the other group operated after 7 weeks (p = 0.03). There was no association between the duration of interval before surgery and postoperative morbidity (complications, transfusion, duration of hospitalization). Kalady et al. studied retrospectively (1997–2007) records of 306 patients operated for a rectal cancer after CRT (50 Gy and 5-fluorouracil) [30]. The dose of radiation received in each group was similar. The rate of pCR (ypT0N0) was 24% in this study. Time between the end of RCT and surgery, with a cut-off estimated at 8 weeks was the single prognostic factor for pCR in uni- and multivariate analysis (OR = 2.63-_95%_CI [1.13 to 6.12], p = 0.02). A. The pathological complete response rate increased from 16.3% (14/86) in the group operated < 8 weeks against 30.8% (28/91) for other (p = 0.03). The authors observed that the period between 8 and 10 weeks showed the greatest number of complete responses and that there was no more gain over 14 weeks. This retrospective study did not give any explanation on the reasons for variations in the time between the end of radiotherapy and surgery [32]. The group of patients with pCR had a better overall survival with a follow-up of 60 months (91% versus 80%, p = 0.046) and less local recurrence at 5 years (0% versus 11%, p = 0.023). A Korean study analyzed retrospectively the data from 12 centers of 306 patients with a tumor classified ypT0 following CRT. This study confirmed the favourable impact of pCR after neoadjuvant CRT on survival: the 5-year overall survival of these patients having 93.4% of tumors initially classified T3 or T4 was 92.8%. Disease-free survival at 5 years was 84.6%. Finally, Kalady et al. compared the outcomes in a retrospective series of 177 patients operated for a rectal cancer after neoadjuvant treatment between patients operated before 8 weeks or after 8 weeks following the end of CRT between 1997 and 2007. Postoperative morbidity or mortality were similar between the two period group. The rate of pCR was lower in the <8 weeks group compared with the > 8 weeks group (16.2% *vs*. 31.1%, p = 0.027). Moreover, the 3 years local recurrence rate was significant lower in the >8 weeks (1.2% vs. 3.9%, p = 0.04) [33].

All these publications are retrospective and the real impact of the delay between the end of CRT and surgery is still a matter of debate [34].

### Aims

The main objective of our study is to evaluate in a randomized trial the impact of a longer interval between the end of CRT and rectal cancer surgery (7 weeks versus 11 weeks) on the rate of pathological complete response. Secondary outcomes include overall and disease free survival, quality of mesorectal excision, rate of sphincter preservation.

## Methods and design

This study is a multicenter randomized open-label controlled trial in parallel groups, comparing two periods between the end of CRT and cancer surgery of rectal tumors: 7 weeks versus 11 weeks. This study has been approved by a national Institutional Review Board: the Regional Comity of Patients Protection of South-West I, N°1-12-19:30/08/12 and by the National Agency of Medicine and Medical Products (ANSM: B111580-10). This study is supported by a grant from the French Ministry of Health (PHRCN 2011, AOM 11314). The research carried out will be on accordance with Helsinki declaration.

### Participants

**Figure 1:**
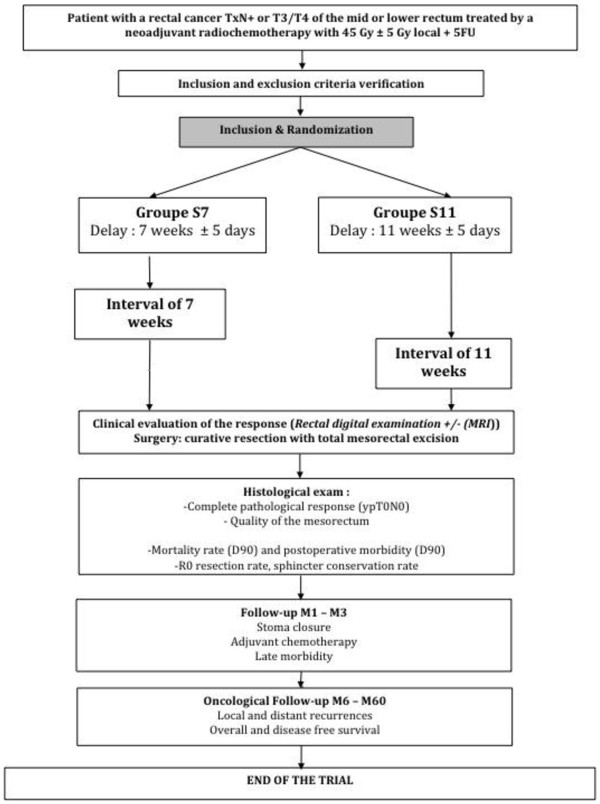
Flow chart.

The institutional promoter is the AP-HP (*Assistance Publique*-*Hôpitaux de Paris* – *DRCD*: *Département de la Recherche Clinique*). Patients are included from several departments of surgery or oncology (n=26) in France (see list of participating centers in the Acknowledgments section). All participating sites signed a convention with the DRCD for ethical approval before beginning of inclusion. All patients must fulfil the following criteria: T3/T4 and/or TxN + mid or low third rectal cancer and completed RCT. The complete inclusion and exclusion criteria are given in Table 1. After oral and written explanation about the purpose of this study, the patient gives his written consent agreeing to participate to the protocol (Figure 1).

**Table 1:** Inclusion and exclusion criteria

**Inclusion criteria**	**Exclusion criteria**
• age over 18 years, no age limit higher	• Patient with metastasis,
• Performance status evaluated by the Eastern Cooperative Oncology Group (ECOG) score: 0-1	• T1 or T2N0 tumor classified by echo -endoscopy and MRI,
	• rectal tumor with lower pole is more than 12 cm from the anal margin or 10 cm from the dentate line,
• Patients with cancer of the middle or lower rectum (lesion located within 10 cm from the dentate line or 12 cm from the anal margin) proved by pathology,	• Patient did not complete the full protocol of radiotherapy,
	• History of tumors (other than basal cell carcinoma and / or carcinoma in situ of the cervix)
• T3-T4N0, TxN+ on ultrasound-endoscopy and MRI, without secondary localization (M0) on the thoraco-abdominal (or chest radiography and abdominal ultrasound)	• A patient with impaired or incompetent
	• investigator by not allowing him a good understanding of the requirements of the study, person under guardianship, persons under guardianship, persons deprived of their liberty by judicial or administrative body, adult subject to legal protection or unable to consent.
• Patient who received a protocol between 45–50 Gy of radiotherapy and chemotherapy based on 5-fluorouracil for an average duration of 5 weeks for the management of rectal cancer,	
• Curative surgical treatment planned following radiochemotherapy with total mesorectal excision,	
• Free and informed consent signed by the patient,	
• Patient affiliated to a social security scheme or beneficiary of such plan (except AME)	
• Patient able, according to the investigator, to comply with the requirements of the study.	

### Randomization

After completion of the pre-intervention assessments, the patients are randomly assigned to the period group (ratio 1:1) by Internet. Blocked centralized randomisation with stratification by centre will be prepared by URC-Est.

### Intervention

#### Surgical resection with total mesorectal excision (TME)

The anaesthesia consultation is planned before the surgery according to the habits of each department. Participation in the study does not alter the anaesthetic procedures. The patient is admitted the day before surgery in the surgical ward.

The type of resection (coloanal anastomosis, abdomino-perineal resection, delayed anastomosis, drainage, laparoscopic approach…) is not influenced by the participation to this protocol. As the rectal tumor is located in the mid- or low rectum a TME is required.

### Pathological exam

The pathological exam required has been already published in the National French Guidelines [35]. A standardized routine pathology examination was performed using the protocol of Quirke et *al*. [36]. After fixation in 10% formalin and inking to assess the circumferential margin, the whole tumor was cut transversely. According to the macroscopical features, different techniques for sampling of tumor tissues were applied in order to avoid any under staging of the specimen:

If there was only ulceration and fibrotic changes without any visible tumoral lesion or if the tumor residual measured less than 3 cm in diameter, the lesion was examined entirely;

If the residual tumor measured more than 3 cm in diameter, one selected block per cm of tumor was processed. If the first selected blocks were free of tumor, complementary blocks were taken and the macroscopically residual lesion was examined in toto. A diagnosis of pCR will only be made after examination of the whole macroscopically residual lesion [37]. The tumor response is evaluated by inclusion of all residual tumor and the response to CRT is graded with the scales of Rodel and Dvorak [22,38]. A double reading of slides will be made for each patient by two independent pathologists blinded to the randomization group of the patient to confirm the ypT stade as the two regression scores. In case of disagreement between the two pathologists, they should jointly give a mutual result.

### Outcomes and assessments

#### Primary outcome

Rate of histological complete response after double reading by two different pathologist.

#### Secondary outcomes

Mobidity, sphincter preservation rate, overall and disease-free survival.

#### Surgical data

During surgery, the operating data are provided on the e-CRF (digital rectal examination under general anaesthesia, type of surgery (anterior resection or abdomino-perineal resection), operative time, intraoperative bleeding, macroscopic appearance of the mesorectum, distance from the distal limit of resection.

#### Morbidity and mortality

The postoperative complications are noted by the surgeon in the e-CRF during hospitalization (about 10–15 days) and during the first 3 months. Postoperative death is defined as death occurring within 30 postoperative days or during the first hospitalization. Postoperative complications are defined by the occurrence of medical or surgical complications within 90 postoperative days or during the first hospitalization. Morbidity will be evaluated with the new classification of surgical complications by Dindo et al. which includes 5 grades [39,40].

#### Pathological exam

Usual data are recorded: distal and circumferiential margins, number of resected and invaded nodes, tumoral differenciation, presence of vascular embols (veinous or lymphatic, intra or extra-mural), perineural engainement, quality of mesorectal excision. The resected specimen will be staged according to American Joint Committee on Cancer (AJCC) criteria (7^th^ version).

#### Rate of sphincter preservation

Comparison between the planned intervention at the time of the randomisation and the resection performed after peroperative digital exam will be performed.

#### Oncological follow-up

Patients will be followed in clinics for 5 years according to the habits of each department. Usual follow-up is composed of clinical exam, CEA analysis, CT-scan or Chest Radiography with abdominal ultra-sound every 3–4 months during the first three years and every 6 months for the last two years.

### Samples size and statistical considerations

With a sample of 264 patients, GRECCAR6 trial has 80% of power to detect at least a two fold increase in the complete response rate in the 11 weeks group compared to the 7 weeks group. This hypothesis assumes that the complete response rate in the 7 weeks group is similar to the usual rate of complete response after 6–8 weeks (12% (Kalady et al. Bosset et al. Hiotis et al.)), with an expected complete response rate in the 11 weeks group of 26% and 10% of drop-outs, using a two-sided test at the 0.05 significance level.

Intention to treat analysis of the primary endpoint will be performed once all randomized patients have 6 months of follow-up. Analysis of survival will be performed after at least 5 years of follow-up. A futility analysis [41] is planned after randomization after 132 patients, using a Bayesian statistical interference, with no impact on Type 1 error [42].

## Discussion

While several retrospective studies has emphasised the role of longer interval on efficiency of radiochemotherapy on histological tumoral response, this multicentric randomized trial is the first to evaluate this factor. The major drawback of previous studies is that no explanations are given on the reasons for variations in the time between the end of radiotherapy and surgery [29,32,43]. Indeed, the time interval was decided by the surgeon or maybe in case of favourable clinical response, mainly authors advocate other factors such as patient morbidity or logistical scheduling issues [33].

This trial could then identify a simple and cheap factor influencing the rate of pCR. While is it still not obvious that pCR is a good suggorate marker of the overall survival [44] based on previous randomized control trial on CRT, it is probable that it could be a prognosis marker of rectal conservation. One publication showed that patients operated after 8 weeks may have significantly less local recurrences.

Indeed, if waiting 4 more weeks increase the rate of pCR, more patients could avoid a morbid surgical procedure such as an abdomino-perineal resection. This new management is still limited due to the actual difficulties to identify patients with pCR before surgery. Adavances in radiological imaging may facilitate this important point.

Finally, this long delay would allow the use of chemotherapy during this 3 months waiting period. This could reduce the risk of synchronous metastasis that may occur during the waiting period.

## Competing interests

The authors declare that they have no competing interests.

## Authors’ contributions

JHL: conception of the study, writing of the manuscript. AR: statistical analysis and help to draft the manuscript. MS: pathological exam and help to draft the manuscript. YP: writing of the manuscript. TS: coordination of the study. ET: conception and coordination of the study. All authors read and approved the manuscript.

## Pre-publication history

The pre-publication history for this paper can be accessed here:

http://www.biomedcentral.com/1471-2407/13/417/prepub
